# Epithelial mesenchymal transition status is associated with anti-cancer responses towards receptor tyrosine-kinase inhibition by dovitinib in human bladder cancer cells

**DOI:** 10.1186/1471-2407-13-589

**Published:** 2013-12-11

**Authors:** Jörg Hänze, Marcus Henrici, Axel Hegele, Rainer Hofmann, Peter J Olbert

**Affiliations:** 1 Department of Urology and Pediatric Urology, Philipps University of Marburg, Baldingerstraße, 35043 Marburg, Germany

**Keywords:** Receptor tyrosine kinase, Tyrosine kinase inhibition, Bladder cancer, Epithelial mesenchymal transition

## Abstract

**Background:**

Dovitinib (TKI-258) is a receptor tyrosine kinase (RTK) inhibitor targeting fibroblast growth factor receptor (FGFR) and further related RTKs. TKI-258 is under investigation as anticancer drug for the treatment of various cancers including bladder cancer with aberrant RTK signaling. Here, we analyzed the responses of ten human bladder cancer cell lines towards TKI-258 treatment in relation to the epithelial mesenchymal transition (EMT) status of the cells.

**Methods:**

Expression of epithelial marker E-cadherin as well as mesenchymal markers N-cadherin and vimentin was determined by quantitative RT-PCR and Western-blot in RNA and protein extracts from the cultured cell lines. The cell responses were analyzed upon addition of TKI-258 by viability/proliferation (XTT assay) and colony formation assay for measurement of cell contact independent growth.

**Results:**

The investigated bladder cancer cell lines turned out to display quite different EMT patterns as indicated by the abundance of E-cadherin or N-cadherin and vimentin. Protein and mRNA levels of the respective components strongly correlated. Based on E-cadherin and N-cadherin mRNA levels that were expressed approximately mutual exclusively, an EMT-score was calculated for each cell line. A high EMT-score indicated mesenchymal-like cells and a low EMT-score epithelial-like cells. Then, we determined the IC_50_ values for TKI-258 by dose response curves (0-12 μM TKI-258) in XTT assays for each cell line. Also, we measured the clonogenic survival fraction after adding TKI-258 (1 μM) by colony formation assay. We observed significant correlations between EMT-score and IC_50_ values (r = 0.637, p = 0.0474) and between EMT-score and clonogenic survival fraction (r = 0.635, p = 0.0483) as analyzed by linear regression analyses.

**Conclusions:**

In sum, we demonstrated that the EMT status based on E-cadherin and N-cadherin mRNA levels may be useful to predict responses towards TKI-258 treatment in bladder cancer.

## Background

Receptor tyrosine kinase (RTK) signaling is altered in urothelial cancer. Namely, FGFR dependent signaling is affected [1]. FGFR3 mutations causing ligand independent dimerization and enhanced kinase activity with constitutive FGFR3 activation are prevalent in low grade non muscle invasive transitional cell carcinoma (TCC) whereas overexpression of wild type FGFR3 is observed in muscle invasive bladder cancer [2-4]. Also, aberrant expression of FGFR1, FGFR2, and FGF2 ligand has been demonstrated [5-7]. Further RTKs such as VEGFR and PDGFR are involved in bladder cancer progression [8]. Therefore, drugs for inhibition of RTKs are under investigation for the treatment of bladder cancer. Among those, TKI-258 targeting signaling of FGFR/PDGFR/VEGFR and further related RTKs is investigated as a potential anti TCC compound [9,10]. The affinity order for TKI-258 has been determined for different RTKs being highest for FGFR1 and FGFR3 followed by VEGFR1-3, PDGFRβ, FLT-3 and c-Kit revealing the complexity of the drug [11]. The responsiveness towards RTK inhibitors is difficult to predict in bladder cancer [7,10]. Patients with non muscle invasive bladder cancer have a good outcome and only a small portion of these tumors progress to metastatic disease. Muscle-invasive TCC is more prone to become metastatic and oncological outcome is much poorer. An indicator of metastatic potential is the EMT status [12]. EMT is associated with enhanced cell migration and metastasis revealing a more aggressive cancer type. Bladder cancer cells can strongly differ in epithelial and mesenchymal characteristics as revealed by different cadherin subtype expression patterns [13,14]. Cadherins are transmembrane cell adhesion proteins that are important during development and play a role in various diseases including cancer. E-cadherin is expressed in epithelial cells. E-cadherin has characteristics of a tumor suppressor that inhibits cell invasion and loss of E-cadherin is important for induction of EMT [15]. During EMT a cadherin switch occurs. E-cadherin is replaced by N-cadherin a well established mesenchymal cell type marker in pathology [14]. P-cadherin is a further cadherin subtype expressed in malignancies but could not yet been assigned to an epithelial or mesenchymal cell type in bladder cancer [14,16]. The mesenchymal marker vimentin represents an intermediate filament that replaces the epithelial cytokeratin filament [17]. The cadherin switch involves transcriptional regulation by epithelial repressors (e.g. snail) for downregulation of E-cadherin and mesenchymal activators (e.g. β-catenin) for upregulation of N-cadherin [18].

Interestingly, unsupervised gene cluster analysis by global gene expression profiling has demonstrated that non-muscle invasive and muscle invasive TCC fall into two distinct subgroups that identified EMT-related genes as relevant [19-21]. The meaning of EMT status for drug responses towards inhibition of epidermal growth factor receptor has been reported in bladder cancer cells and revealed a relevance of E-cadherin expression [22,23].

Here, we characterized ten human bladder cancer cell lines with respect to expression of E-cadherin, N-cadherin and vimentin. Furthermore, we analyzed the response of these cells towards treatment with TKI-258 by proliferation/viability assay and colony formation assay. We observed that cells with epithelial characteristic had a stronger therapeutically beneficial response to TKI-258 than cells with mesenchymal characteristics. Thus, analysis of the EMT status may help to predict TKI-258 responsiveness independent of molecular analysis of RTK signaling.

## Methods

### Cell culture

Human bladder cancer cell lines T24, HT1376, BFTC-905, 5637, HU456, UMUC3, RT4, RT112, TCC-SUP, MGHU4 were cultured in RPMI1640 medium supplemented with 10% fetal bovine serum, 1% stable glutamine and 1% Penicillin/Streptomycin solutions (PAA Laboratories, Pasching, Austria) at 37°C with 5% CO_2_ in humidified air. Dovitinib (TKI-258) was kindly provided by Novartis Pharma AG (Basel, Switzerland). RT4 and RT112 cells are known to be wild type for FGFR3 and T24 and UMUC3 have activating RAS mutations acting downstream of RTKs [10].

### RNA and protein extraction

RNA and protein extraction was performed with Trifast (Peqlab, Erlangen, Germany) according to the manufacturer’s protocol.

### Quantitative real time RT-PCR

1 μg RNA was used as template for cDNA synthesis after digest of genomic DNA with RNase-free DNase (RevertAid First Strand cDNA synthesis Kit, Fermentas Life Science, St. Leon-Rot, Germany). Realtime RT-PCR was performed with SYBR Green Fluorescein Mix (ABgene UK, Epsom, UK). Cycling conditions were, 95°C for 15 min, followed by 45 cycles of 95°C for 15 s, 60°C for 15 s, 72°C for 30 s. Relative levels of mRNA are displayed as -ΔCt values with the mean of β-actin and porphobilinogen deaminase (PBGD) as reference mRNA (ΔCt = Ct_target mRNA_ – Ct_reference mRNA_). The following primer sets (+, forward; -, reverse) were employed: N-cadherin (+: CAA TCC TCC AGA GTT TAC TGC CAT G, -: GAT TGG TTT GAC CAC GGT GAC TAA C), E-cadherin (+: TGA AAA GAG AGT GGA AGT GTC CGA G, -: GAT TAG GGC TGT GTA CGT GCT GTT C), P-cadherin (+: GAC ACC TTC CGA GGG AGT GTC TTA G, -: GGA TGG TCA GTG TGT ACT CAG GGA C), Vimentin (+: GCA AAG CAG GAG TCC ACT GAG TAC C, - TGT CAA GGG CCA TCT TAA CAT TGA G), PBGD (+: TGT CTG GTA ACG GCA ATG CG, -: CCC ACG CGA ATC ACT CTC AT), FGFR3 (+: GAA AGA CGA TGC CAC TGA CAA GGA C, -: TCC TTG AAG GTG AGC TGC TCC TC), β-actin (+: TAT CCA GGC TGT GCT ATC CCT GTA C, -: TTC ATG AGG TAG TCA GTC AGG TCC C) (Biomers GmbH, Ulm, Germany). The EMT-score was defined as (-Δct N-cadherin) – (-Δct E-cadherin).

### Western blot

After determination of protein concentration (Pierce BCA Protein Assay, Thermo Scientific, Rockford, USA), 40 μg of each sample was subjected to sodium dodecyl sulphate polyacrylamide gel electrophoresis (10% polyacrylamide) and transferred to polyvinylidene fluoride membrane (Millipore, Bedford, USA) by electrophoresis. The membranes were blocked at room temperature for 1.5 h. Primary antibodies for vimentin (dilution 1:2500), E-cadherin (dilution 1:5000), N-cadherin, (dilution 1:2000) (all of these polyclonal rabbit antibodies (Abgent, San Diego, USA) and for β-actin (dilution 1:5000; monoclonal mouse Abcam, Cambridge, UK) were added and incubated overnight at 4°C in tris-buffered saline with 0.1% tween containing 5% dry milk. Then, secondary horseradish peroxidase coupled anti-rabbit or anti-mouse immunoglobulin (both dilution 1:2000; Thermo Scientific, Rockford, USA) was added for band detection with enhanced chemiluminescent luciferase kit (Thermo Scientific, Rockford, USA) by an image system (Fluorchem IS-8900, Alpha Innotech, San Leandro, USA) allowing measurement of band intensity for determination of relative protein abundance.

### Proliferation/viability assay

*TACS XTT-Kit* (Trevigen, Gaithersburg, USA) with a long term protocol was used to assess the effects of TKI-258 on cell viability, an assay that closely correlates with proliferation. Cells were seeded into 96-well plates with 150 μl medium and TKI-258 was added one day later in a dose range as indicated (0.25 μM – 12 μM). Medium and TKI-258 was replaced once after 2 d and incubation continued for further 3 d. Then, XTT solution was added and the optical density was measured at 490 nm. The IC_50_ values were calculated by non-linear regression analysis with the equation of a sigmoidal dose response with variable slope (Graphpad Prism 5.0): Y = 1/[1 + 10^(logIC50 ‒ X)(Hillslope)].

### Colony formation assay

This assay measures cell proliferation in a cell contact independent way. Cells were plated in pre-tested appropriate densities yielding 100-500 cells per plate. The plates were cultured for 8-12 days in the presence (1 μM) or absence (0 μM) of TKI-258. Then, the colony signals were densitometrically measured after crystal violet staining. The clonogenic survival fraction was defined as the ratio of signal intensity of untreated group versus TKI-258 (1 μM) treated group.

## Results

**Figure 1:**
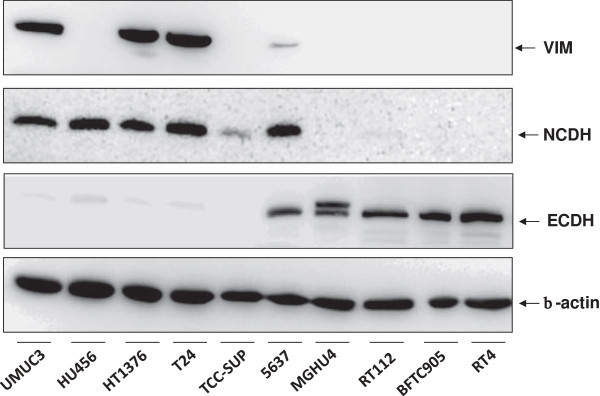
**Western-blot analysis of mesenchymal markers vimentin (VIM) and N-cadherin (NCDH) as well as epithelial marker E-cadherin (ECDH) in comparison to cytoplasmic β-actin in various human bladder cancer cell lines indicated at the bottom.** It is obvious that cells strongly differ in the expression levels of VIM, NCDH and ECDH indicating mesenchymal-like or epithelial-like cell characteristics.

**Figure 2:**
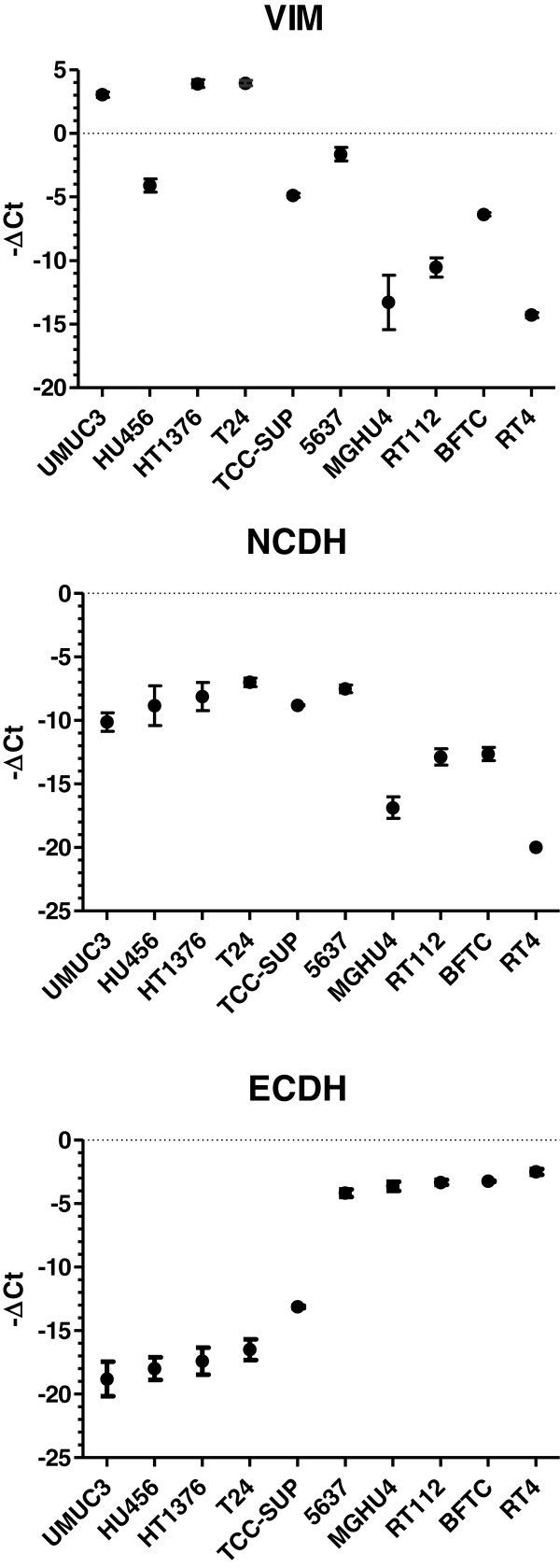
**Quantification of mRNA encoding vimentin (VIM), N-cadherin (NCDH) and E-cadherin (ECDH) by realtime RT-PCR in human bladder cancer cell lines.** Displayed are the -ΔCt values (Ct, cycle of threshold) normalized to β-actin and PBGD mRNA (mean, standard deviation, n ≥ 3). The order of cell lines is the same as in the Western-blot and allows direct comparison with Figure 1. Linear regression analysis revealed strong correlation between mRNA and protein levels of NCDH, ECDH and VIM, respectively (E-cadherin, r = 0.831, p = 0.0029; N-cadherin, r = 0.794, p = 0.0061; vimentin, r = 0.858, p = 0.0015).

We analyzed typical components indicating the epithelial or mesenchymal cell status in ten human bladder cancer cell lines. As epithelial marker we measured E-cadherin and as mesenchymal markers N-cadherin and vimentin by Western blot (Figure 1). E-cadherin and N-cadherin expression levels appeared almost mutually exclusive and vimentin was predominantly expressed in those cells that were N-cadherin positive. Next, we quantified the mRNA levels of these components (Figure 2). We revealed strong correlation between mRNA and protein levels suggesting major regulation of these components at the mRNA level.

**Figure 3:**
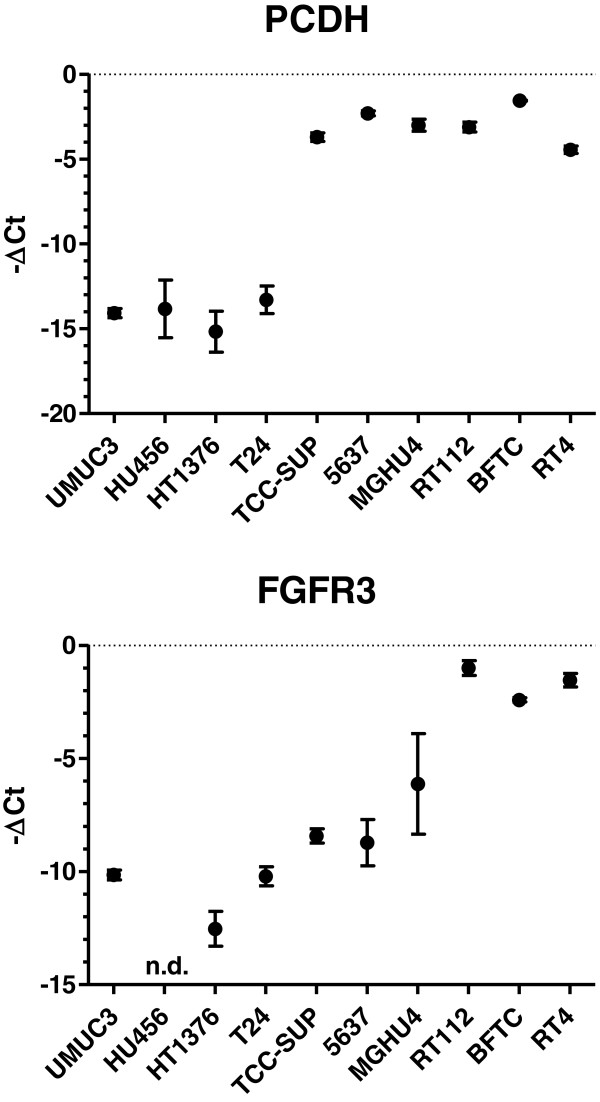
**Quantification of P-cadherin (PCDH) and fibroblast growth factor receptor 3 (FGFR3) mRNAs by realtime RT-PCR in human bladder cancer cell lines.** Displayed are the -ΔCt values (Ct, cycle of threshold) normalized to β-actin mRNA and PBGD mRNA (mean, standard deviation, n ≥ 3).

In addition, we analyzed P-cadherin and FGFR3 (Figure 3). The role of P-cadherin has been ambiguously described in EMT status. FGFR3 was analyzed since FGFR3 was demonstrated to correlate with epithelial markers. Interestingly, we revealed a correlation between P-cadherin and E-cadherin-mRNA levels (r = 0.919, p < 0.0001) and could confirm the correlation between FGFR3 and E-cadherin-mRNA (r = 0.813, p < 0.0001).

**Figure 4:**
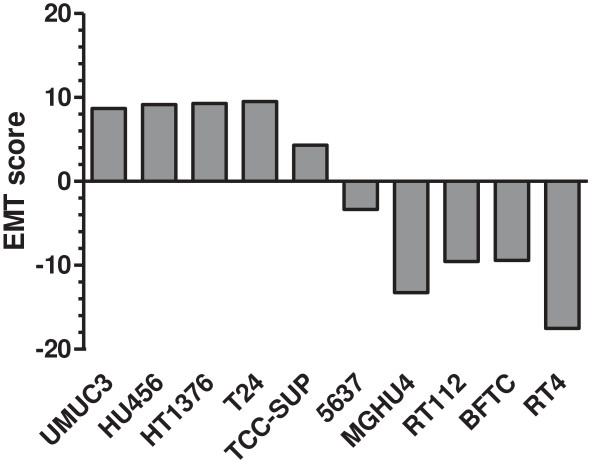
**Calculation of an EMT-score for each bladder cancer cell line by subtraction of –Δct [NCDH] - –Δct [ECDH] (data from Figure **2**).** This score allows quantitative evaluation of mesenchymal (high values) and epithelial (low values) characteristics.

**Figure 5:**
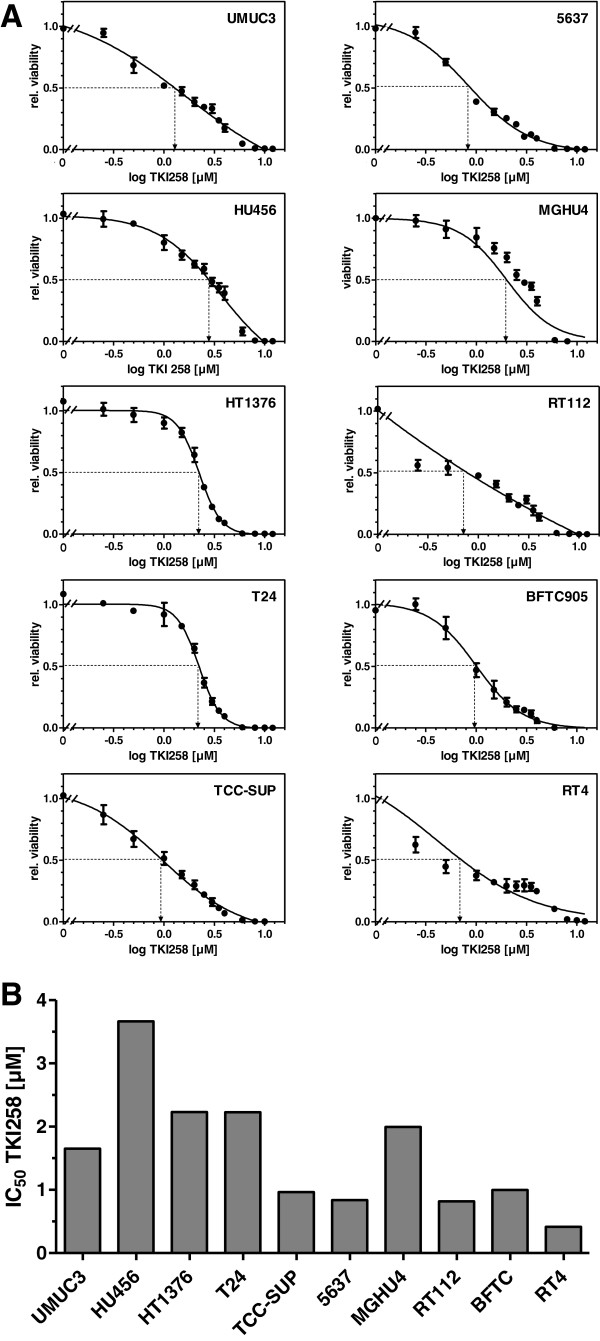
**Calculation of IC50 values for TKI-258 treatment. A)** Proliferation/viability XTT assay in human bladder cancer cell lines. The IC50 values were determined by curve fitting with non-linear regression analysis (sigmoidal dose response). **B)** The IC50 values for each cell line are summarized.

**Figure 6:**
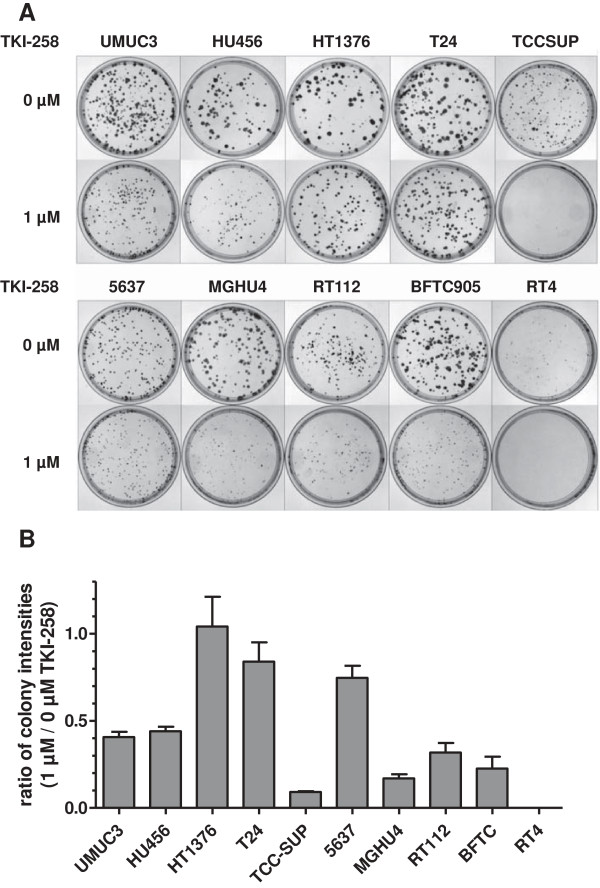
**Calculation of the clonogenic survival fraction after TKI-258 treatment. A)** Colony formation assay of control cells TKI-258 [0 μM] and cells treated with TKI-258 [1 μM]. Displayed are the plates after staining with crystal violet. **B)** The ratio of signal intensity of the plates treated with TKI-258 [1 μM] versus control [0 μM] is displayed indicating the clonogenic survival fraction as a quantitative parameter of treatment efficiency with TKI-258.

**Figure 7:**
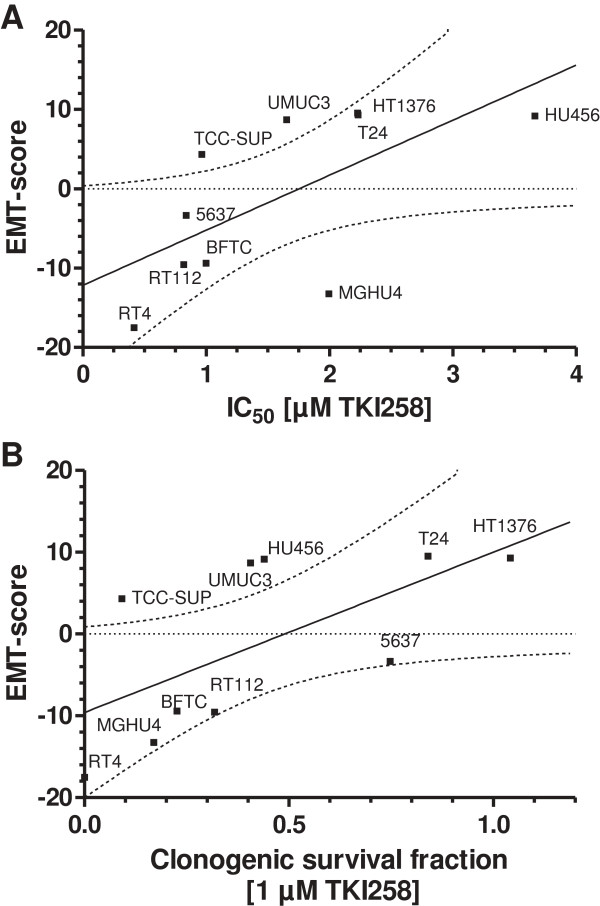
**Linear correlation analyses of the EMT-score with TKI-258 responses. A)** Correlation of EMT-score and IC50 values (r = 0.637, p = 0.0474). **B)** Correlation of EMT-score and clonogenic survival fraction (r = 0.635, p = 0.0483). The dotted lines represent the 95% confidence band of the best fit, respectively.

Based on the well established and related endpoint markers of EMT status, E-cadherin and N-cadherin, we calculated an EMT-score for each cell line by subtraction of -ΔCt N-cadherin and -ΔCt E-cadherin, respectively (Figure 4). In this term, high values reflect a mesenchymal status and low values an epithelial status. Based on this EMT-score, we analyzed the cell responses towards TKI-258 treatment. Employing a proliferation/viability assay, we measured the inhibitory concentration of TKI-258 yielding 50% viable cells (IC_50_ value) by establishing dose response curves for each cell line (Figure 5A, B). Furthermore, we performed colony formation assay for the measurement of cell contact independent growth. We determined the clonogenic survival fraction by calculating the ratio of cells treated with TKI-258 (1 μM) compared to untreated control (0 μM) (Figure 6A, B). These data were analyzed by linear regression analyses between the EMT-score (data from Figure 4) and the IC_50_ value (data from Figure 5B) and between the EMT-score and the clonogenic survival fraction (data from Figure 6B). We observed significant correlations between EMT-score and IC_50_ values (Figure 7A) and between EMT-score and clonogenic survival fractions (Figure 7B).

In conclusion, the EMT status as determined by E-cadherin and N-cadherin mRNA levels demonstrated significant correlation with cellular TKI-258 responses as studied by different experimental approaches in bladder cancer cell lines.

## Discussion

The EMT status reflects features of cancer cells that favor cell migration and invasion, characteristics that are linked to metastasis. Epithelial-like cells are crucially characterized by E-cadherin and mesenchymal-like cells by N-cadherin expression. In cancer, the EMT status reflects the issue of complex cell signaling mechanisms including RTK pathways [24]. Aberrant signaling of RTKs has been described in bladder cancer [25]. Therefore, TKIs are studied for therapy of bladder cancer however, the therapeutic responses vary and are difficult to predict.

Here, we investigated the EMT status in bladder cancer cell lines and tested whether the EMT status is associated with therapeutic responses towards TKI-258. Most importantly, we demonstrated: 1) E-cadherin and N-cadherin protein levels were expressed complementary and correlated with their respective mRNA levels. 2) N-cadherin and E-cadherin mRNA levels served for calculation of an EMT-score indicating the EMT status. High values reflected a relative mesenchymal cell type and low values an epithelial-like cell type. 3) Analysis of the EMT-score and cell responses towards TKI-258 treatment revealed correlations that indicated epithelial-like cells as more therapeutically responsive than mesenchymal-like cells.

Beside the well defined role of E-cadherin and N-cadherin in EMT, we also included P-cadherin in our studies. We observed striking correlation of P-cadherin and E-cadherin mRNA levels supporting a possible association of P-cadherin with epithelial characteristics. This finding is in line with studies where P-cadherin was observed to be enhanced in low grade non muscle invasive bladder cancer indicating epithelial differentiation [16,26]. Other studies revealed correlation of P-cadherin levels with increasing tumor and grading stage indicating a mesenchymal characteristic [14,27,28]. In contrast, the role of N-cadherin and E-cadherin in EMT is clearly defined. Therefore, calculation of an EMT-score based on these cadherin subtypes appeared reasonably and revealed correlations with TKI258 responses in all cell assays performed.

Noteworthy, RTK signaling is related to the expression of epithelial and mesenchymal markers. In particular, FGFR3 mRNA correlated with E-cadherin mRNA [7] as confirmed in the cell lines in our study. Furthermore, FGFR1 mRNA expression (not investigated in our study) correlated with the mesenchymal marker N-cadherin [7]. Thus, the analysis of the EMT may be an alternate clue to predict responses towards inhibition of RTK signaling in cancer cells without the need to identify possible aberrations of RTK or downstream components by molecular diagnostics. Noteworthy, prediction of cellular responses towards TKI-258 solely based on mutation studies of FGFR have failed and the identification of superior biomarkers is desirable [10]. The analysis of EMT parameters as performed in our study in human cancer cell lines would be also applicable for tumor tissue samples.

Restrictively, it has to be addressed that TKI-258 targets several RTKs namely those of the ligands VEGF, PDGF and FGF that represent growth and angiogenic factors [1,29]. Thus, in vivo effects of TKI-258 are certainly more complex and comprise effects on tumor angiogenesis. Moreover, effects of TKI-258 have not only been attributed to inhibition of RTKs. Namely, topoisomerase II has been demonstrated as target of TKI-258 causing cytotoxic DNA double strand breaks [30].

## Conclusions

Aberrant cellular processes that contribute to bladder tumorigenesis comprise altered signaling of RTKs. Thus, tyrosine kinase inhibitors such as TKI-258 are under investigation for the treatment of bladder cancer. Here we demonstrated that the EMT status determined by E-cadherin and N-cadherin expression levels is associated with responses towards TKI-258 treatment. In particular, TKI-258 was more effective in epithelial-like than in mesenchymal-like bladder cancer cells. Therefore, determination of the EMT status may be exploited as putative predictor for treatment responses of TKI-258 in bladder cancer.

## Competing interests

The authors declare that they have no competing interests.

## Authors’ contributions

Conception and design: JH, MH, AH, RH, PJO. Development of methodology: MH, JH. Acquisition of data: MH, JH. Analysis and interpretation of data: JH, MH. Writing and reviewing of the manuscript: JH, MH, PJO. All authors read and approved the final manuscript.

## Pre-publication history

The pre-publication history for this paper can be accessed here:

http://www.biomedcentral.com/1471-2407/13/589/prepub
